# The MBS microbial rapid detection system for rapid detection of major pathogenic bacteria in feed: comparison with plate counting method

**DOI:** 10.1186/s12866-022-02655-2

**Published:** 2022-10-06

**Authors:** Linlin Jiang, Beibei Zhang, Shuitao Liu, Lianqin Zhu, Fenghua Zhu

**Affiliations:** 1 College of Veterinary Medicine, Qingdao Agricultural University, Qingdao, People’s Republic of China; 2 College of Animal Science and Technology, Qingdao Agricultural University, Qingdao, People’s Republic of China

**Keywords:** MBS microbial rapid detection system, PCM, Feed, Pathogenic bacteria

## Abstract

The current methods for detecting pathogenic bacteria in feed require high technique and take a long time. The Micro Biological Survey (MBS) rapid detection system is a simple, economical and rapid microbial detection method. The purpose of this experiment was to compare the detection of *Escherichia coli* (*E. coli*), *Salmonella*, *Staphylococcus aureus* (*S. aureus*), *Listeria monocytogenes* (LM), coliform (COLI) and total viable count (TVC) in feed by the MBS rapid microbial detection system and plate counting method (PCM). The results showed that the limit of quantitation, recovery rate and coefficient of variation of the MBS microbial rapid detection system are better than the plate counting method. When detecting the pathogenic bacteria content in artificially contaminated feed, the MBS rapid microbial detection system was positively correlated with the PCM. When the MBS microbial rapid detection system and PCM were used to detect the collected real feed samples, there was no significant difference in the detection results of the two methods in most of the feed samples. In summary, the MBS microbial rapid detection system is the most convenient and rapid detection method and is suitable for promotion and application in production lines.

## Introduction

The pathogenic bacteria in feed come from the external contamination of feed production, storage, transportation and sales, one of the main factors affecting feed safety. There are many types of pathogenic bacteria in feed, including pathogenic *Escherichia coli* (*E. coli*), *Salmonella*, *Clostridium botulinum*, *Shigella*, and *Staphylococcus aureus* (*S. aureus*). Pathogens can infect livestock and poultry through feed, causing significant economic losses to the breeding industry. The contamination degree of various pathogens varies greatly depending on the type of feed, among which *E. coli, Salmonella, S. aureus* and LM are more harmful. *E. coli* is a Gram-negative bacterium, the most representative serotype is O157:H7, which can cause hemorrhagic enteritis and diarrhea in humans and animals [1–3]. *Salmonella* is also a Gram-negative bacterium, and the pollution rate of *Salmonella* in livestock and poultry feed is very high [4]. Studies have found that different serotypes of *Salmonella* exist in feed ingredients, feed and production workshops [5, 6]. As one of the main pathogenic bacteria of humans and animals, *Salmonella* can cause diseases such as pullorum disease, paratyphoid fever in piglets, and animal abortion. And *Salmonella* can also cause gastroenteritis, typhoid fever, sepsis, extraintestinal focal infection, food poisoning and other syndromes in humans [7]. *S. aureus* is a Gram-positive bacterium, which is a typical zoonotic pathogen and can cause local purulent infections [8]. There are many ways of contamination of *S. aureus* in feed [9]. *Listeria monocytogenes* (LM) is a Gram-positive bacterium and grows best in a neutral or slightly alkaline environment [10, 11]. LM has been detected in the feces of 5% of healthy people and in many foods [12]. LM often causes fatal infections of the blood and central nervous system [13] and can also cause listeriosis in humans and animals, mainly manifested as meningitis, sepsis and abortion [11, 14].

China’s “Feed Hygiene Standard” stipulates that TVC detected in animal-derived feed should be < 2 × 10^6^ CFU/g, and pathogenic bacteria such as *Salmonella* should not be detected in 25 g samples [15].

At present, there are many detection methods for pathogenic bacteria in feed, such as the traditional microbiology-based plate counting method, which is time-consuming and labor-intensive and has complicated steps; polymerase chain reaction, etc., all require professional technicians and laboratories. The MBS microbial rapid detection system is a convenient, fast and efficient method for the detection of microorganisms [16]. This method is based on an internationally leading microbial detection instrument that integrates the Petri dish method, enzymatic method, immune method and genetic method. The MBS Microbial Rapid Detection System measures the catalytic activity of oxidoreductases in the main metabolic pathways of microorganisms through redox indicators [17–19]. If the target microorganism is present, the redox-reactive pigment in the test vial changes color depending on the redox state of the medium. The MBS host detects the color change through three light waves and finally determines the microorganism content according to the time of integrating the color change. The time taken for color change is inversely correlated with the log value of the degree of microbial contamination, allowing a definite correlation between the observed enzymatic activity and the number of viable cells in the test sample. The more microorganisms there are, the shorter the time required for color change; the fewer microorganisms there are, the longer the time required for color change; if the target microorganism does not exist, the color of the reagent will not change [20].

To date, MBS rapid microbial detection technology has been widely used in water quality [21], food and other microbial detection. Its detection range includes the total number of viable bacteria (TVC) and coliforms (COLI), *E. coli, Enterobacteriaceae*, fecal coliforms, *S. aureus, Salmonella*, and *Listeria monocytogenes* (LM) [22]. Losito et al. [22] tested different forms of water samples in Eritrea, including bottled water, tap water from public distribution systems, domestic water, and river water. Finally, the results obtained by the MBS microbial rapid detection system showed that except for the number of microorganisms in the bottled water that met the drinking water standard, the water quality of other sources did not meet the standard. Bottini et al. [23] detected the total number of bacteria and coliforms in cheese, meat, vegetables, fruits and other foods, compared them with international standard reference methods, and found that MBS microbial rapid detection technology can be used to detect the total number of bacteria and the number of coliform bacteria in the food industry.

The MBS microbial rapid detection system can detect 8 different items at one time in 13 different standard culture environments, has simple operation, high sensitivity, and strong specificity and can be fully automated. At present, this technology is responsible for microbial detection in most laboratories in Europe, and its sensitivity complies fully with EU and domestic standards.

There is no relevant research or report on this method for detecting pathogenic bacteria in feed at this stage. Therefore, this study compares the detection results of the main pathogens in artificially contaminated feed through the MBS rapid microbial detection system and the PCM to evaluate the sensitivity, accuracy and precision of the MBS microbial rapid detection system and the linear relationship between the two methods to provide a scientific basis for the future application of this method to the detection of pathogens in feed.

## Materials and methods

### Strain

Standard strains of *Salmonella* (CVCC2220), *Staphylococcus aureus* (CVCC4098), *Listeria monocytogenes* (CVCC1597), *Escherichia coli* (CVCC1299) and *Klebsiella pneumoniae* (CVCC4079) were provided by the China Veterinary Culture Collection Center. Standard strains of *Citrobacter freundii* (ATCC43864) and *Enterobacter cloacae* (CMCC43501) were provided by BeNa Culture Collection Co., Ltd.

### Sampling

The feed ingredient samples, including blood meal, meat and bone meal, fish meal, feather meal and whey meal, and mixed feed samples, including laying hen complete feed, broiler complete feed, pig complete feed and meat duck complete feed, were randomly collected from feed-producing companies and animal farms across the Shandong Province of China; additionally, 5 different samples were collected per feed. A total of 500 g each of fish meal, meat and bone meal and broiler complete feed was placed in a conical flask, sterilized by high-pressure steam at 121 °C for 20 min, dried and set aside. There were 9 kinds of feed, 5 parts of each feed, for a total of 45 feed samples. Feed samples were collected according to the sampling method of GB/T 14,669.1–2005 [24]. The sampling process followed aseptic operating procedures to prevent all external contamination. The collected samples were placed in a foam box containing an ice bag, transported to the laboratory as soon as possible, and stored at -20℃ for further analysis.

### Resuscitation, cultivation and preservation of strains

Seven strains were recovered in strict accordance with the method recommended by the China Veterinary Culture Collection Center. The 7 resurrected standard strains were streaked and inoculated on nutrient agar solid medium. They were cultured at 37 °C for 12–18 h. A single colony was picked and placed in 100 mL trypticase soy broth, placed in a constant temperature shaker for 120 r/min and incubated overnight at 37 °C for later use. One milliliter of the bacterial liquid in the logarithmic phase was added to a sterile cryotube containing 40% glycerol and stored at -70℃ ultra-low temperature refrigerator for later use.

### Preparation of artificially contaminated samples

The bacterial suspensions of the 7 standard strains were separately cultured in tryptic soy broth (TSB) overnight and then mixed in equal amounts to prepare a standard bacterial solution for the total number of bacteria. The bacterial suspensions of the 4 standard strains (*E. coli*, *Citrobacter freundii*, *Enterobacter cloacae*, and *Klebsiella pneumoniae*) were separately cultured in TSB overnight and then mixed in equal amounts to prepare a standard bacterial solution for COLI. Bacterial suspensions of *Salmonella*, *S. aureus*, LM, and *E. coli* were subjected to TSB enrichment culture. Six standard bacterial solutions were used to prepare bacterial solution samples with different dilutions (10^0^ ~ 10^8^ CFU/ml, dilute of standard bacterial solution by doubling dilution method, then count the plate and calculate the concentration of the bacterial solution.) by the serial dilution method. Five milliliters of bacterial solution were sampled from each dilution, placed in a 50 mL sterile centrifuge tube containing 5 g of sterile fish meal (or meat and bone meal, broiler complete feed), mixed and stored at 4 ℃. When using PCM for detection, it is necessary to dilute the sample homogenate with accurate and quantitative sterile saline and allowed to stand for 10 min (in an environment of 2–4 °C), and then the supernatant was drawn for separation, culture and counting. When using MBS for detection, directly weigh 1 g of sample homogenate and perform the detection according to the steps.

### Determination by MBS

Table 1 shows the incubation temperature and detection schedule for the rapid microbiological test (MBS). Ten milliliters of sterile glycerol matching the reagent bottle in the MBS microbial rapid detection system was placed into a reagent bottle (in the standard set). Next, 1 g (accurate to 0.0001 g) of feed was placed into a reagent bottle and agitated for 20 s on a vortex shaker. After the MBS rapid microbial detection analyzer was turned on, the reagent bottle was placed into the instrument for incubation. After setting the parameters, the MBS could automatically be detected and analyzed. The experiment was repeated 5 times for each sample. After the analysis was completed, the top of the reagent bottle was pressed, and the disinfection and sterilization substances inside the bottle cap were released into the reagent bottle, which could be fully sterilized in 5 ~ 10 min. The sterilized reagent bottle was discarded into the biological waste bin for centralized processing. After the test, the system can output the test report. The content of the report includes all the information set by the user, the test results, such as the discoloration time, the concentration of microorganisms in the sample and all the parameters in the test.

**Table 1:** Microbial rapid test (MBS) incubation temperature and test schedule

**Strains**^**a**^		**TVC**	**COLI**	E.***coli***	***S. aureus***	***Sal***	***Listeria***
**Incubation temperature (℃)**		30	37	37	37	37	37
**-**		BLUE	RED	RED	RED	RED	BLUE
** + **		YELLOW	YELLOW	YELLOW	YELLOW	YELLOW	YELLOW
**bacterial concentration**	**CFU/ml or CFU/g**	**Discoloration time(h)**					
	**1 × 10**^**7**^	< 3.0	< 4.0	< 4.3	< 7.3	< 4.0	< 7.3
	**1 × 10**^**6**^	3.0	4.0	4.3	7.3	4.0	7.3
	**1 × 10**^**5**^	5.3	6.3	7.3	11.0	4.3	12.0
	**1 × 10**^**4**^	8.0	9.3	10.0	20.0	8.3	16.3
	**1 × 10**^**3**^	11.0	12.3	13.0	29.3	13.3	20.3
	**1 × 10**^**2**^	14.0	16.0	16.0	36.0	18.0	25.0
	**1 × 10**^**1**^	16.0	19.0	19.0	43.0	23.0	29.0
	**1**	18.0	22.0	22.0	46.0	28.0	33.0
	**0**	> 20.0	> 24.0	> 24.0	> 48.0	> 32.0	> 36.0

^a^*E. coli* Escherichia coli*, Sal,* Salmonella*, S. aureus* Staphylococcus aureus*, Listeria* Listeria monocytogenes, *COLI* Coliforms, *TVC* Total viable count

### Determination by PCM

Then, 25 g (accurate to 0.0001 g) of ground feed was placed into a sterile flask, and 225 ml of phosphate buffer solution was added to the flask. Next, the solution was shaken for 5 min on a mechanical shaker. After standing for 5 min, the solution was made into a tenfold diluted sample homogenate, and a tenfold serial dilution was performed. The sample homogenate was diluted with accurate and quantitative sterile saline and allowed to stand for 10 min (in an environment of 2–4 °C), and then the supernatant was drawn for separation, culture and counting. The experiment was repeated 5 times for each sample. *E. coli, Salmonella, S. aureus,* LM, COLI, TVC, respectively, in accordance with the National Criterion of China GB/T 4789.38–2012 [25], GB/T 13,091–2018 [26], GB/T 4789.10–2016 [27], GB/T 4789.30–2016 [28], GB/T 18,869–2019 [29] and GB/T 13,093–2006 [30] were used for plate culture and counting.

## Method validation

### The limit of quantitation

The fish meal contaminated with 6 standard bacterial solutions at different dilutions (10^3^, 10^2^, 10^1^, 10^0^ cfu/mL) was chosen, diluted with accurate and quantitative sterile normal saline, and allowed to stand for 10 min. Next, the supernatant was aspirated, and bacteria were plate-cultured and counted according to the National Criterion of China. At the same time, the fish meal contaminated by the bacterial suspension at each dilution was tested according to the operating method of the MBS rapid microbial detection system. The fish meal contaminated by the bacterial suspension of each dilution was tested in 3 parallels, and then the quantitation limits of the two methods were recorded.

### Recovery studies

Nine fish meal samples contaminated with 6 standard bacterial solutions of different dilutions (10^8^, 10^7^, 10^6^, 10^5^, 10^4^, 10^3^, 10^2^, 10^1^, 10^0^ cfu/mL) were chosen, diluted with accurate and quantitative sterile normal saline, and allowed to stand for 10 min. Next, the supernatant was aspirated, and bacteria were plate-cultured and counted according to the National Criterion of China. At the same time, the fish meal contaminated by the bacterial suspension of each dilution was tested according to the operation method of the MBS rapid microbial detection system. The fish meal contaminated by the bacterial suspension of each dilution was tested in 3 parallels. The recovery rate was expressed as the deviation of experimental from nominal concentration values in percent, recovery (%) = (measured value/theoretical value) × 100.

### Coefficient of variation

The fish meal contaminated with 6 standard bacterial solutions of 10^5^ dilution was chosen, diluted with accurate and quantitative sterile normal saline, and allowed to stand for 10 min. Next, the supernatant was aspirated, and 12 parallel samples were prepared for each standard bacterial solution and counted according to the National Criterion of China. At the same time, samples were tested according to the operating method of the MBS rapid microbial detection system. Coefficient of variation (C. V.) = (standard deviation (SD)/‾X) × 100%

### Real sample determination

The content of *E. coli, Salmonella, S. aureus,* LM, COLI and TVC in the samples (blood meal, meat and bone meal, fish meal, feather meal, whey meal, compound feed for laying hens, compound feed for broilers, compound feed for pigs, compound feed for meat ducks) were determined by an MBS microbiological rapid detection system and PCM, respectively. The mean, median and maximum pathogenic bacteria concentrations in the feed samples detected by the two methods are listed in Tables 6 and 7. The median is a more representative parameter than the mean and standard deviation when the distribution of data presents a positive skew. Additionally, to show the most serious pathogen contamination, we listed the maximum value. These two parameters are beneficial for the statistical description of the data.

### Correlation between the two methods

Three feed samples (fish meal, meat and bone meal and broiler complete feed) contaminated with 6 standard bacterial solutions of different dilutions (10^8^, 10^6^, 10^4^, 10^2^, 10^0^ cfu/mL) were chosen, diluted with accurate and quantitative sterile normal saline, and allowed to stand for 10 min. Next, the supernatant was aspirated, and bacteria were plate-cultured and counted according to the National Criterion of China (GB/T 18,869–2019) [18]. At the same time, the sample contaminated by the bacterial suspension of each dilution was tested according to the operating method of the MBS rapid microbial detection system. The sample contaminated by the bacterial suspension of each dilution was tested in 5 parallel experiments.

### Statistical analysis

SPSS statistical software (version 26.0, IBM Corp., Chicago, IL, USA) was used for statistical and linear regression analysis. Differences in the limit of quantification, recovery rates and coefficient of variation of pathogenic bacteria in feed measured by the MBS rapid microbial detection system and PCM methods were analyzed by one-way analysis of variance (ANOVA) with a post hoc Duncan’s multiple comparisons test. The limit of quantification, recovery rates and coefficient of variation results are presented as the mean ± standard deviation (SD).

## Results and discussion

### Comparison of sensitivity between the MBS microbial rapid detection method and PCM

The quantification limits of the PCM and MBS rapid microbial detection systems are shown in Table 2. The minimum limits of quantitation (LOQs) of *E. coli, Salmonella, S. aureus,* LM, COLI and TVC by the plate count method were 41.30 cfu/mL, 56.70 cfu/mL, 39.00 cfu/mL, 56.00 cfu/mL, 40.00 cfu/mL and 48.00 cfu/mL, respectively. The LOQs of *E. coli, Salmonella, S. aureus,* LM, COLI and TVC by the MBS Microbial Rapid Detection System were 5.58 cfu/mL, 8.60 cfu/mL, 7.90 cfu/mL, 8.40 cfu/mL, 3.93 cfu/mL and 7.43 cfu/mL, respectively. Gionfriddo et al. [21] used the MBS microbial rapid detection method to detect coliforms in different water sources, and the quantification limit was lower than 10 cfu/mL, which was similar to our results. The LOQs of the MBS rapid microbial detection system were lower than the LOQs of the plate counting method.

**Table 2:** Comparison of quantification limits of six standard strains by two methods

Strains^a^	Bacteria concentration (cfu/mL)	PCM (cfu/mL)	MBS (cfu/mL)
E.***coli***	10^3^	(3.94 ± 0.31) × 10^3^	(3.77 ± 0.76) × 10^3^
	10^2^	(3.99 ± 0.51) × 10^2^	(4.46 ± 1.17) × 10^2^
	10^1^	(4.13 ± 0.15) × 10	(3.96 ± 0.65) × 10
	10^0^	0	5.58 ± 0.49
***Sal***	10^3^	(2.32 ± 0.35) × 10^3^	(1.80 ± 0.26) × 10^3^
	10^2^	(4.27 ± 2.06) × 10^2^	(2.17 ± 0.28) × 10^2^
	10^1^	(5.67 ± 0.35) × 10	(4.97 ± 6.05) × 10
	10^0^	0	8.60 ± 0.85
***S*.*aureus***	10^3^	(1.55 ± 0.06) × 10^3^	(1.74 ± 0.30) × 10^3^
	10^2^	(1.59 ± 0.05) × 10^2^	(1.85 ± 0.25) × 10^2^
	10^1^	(3.90 ± 0.30) × 10	(6.27 ± 0.47) × 10
	10^0^	0	7.90 ± 1.93
***Listeria***	10^3^	(3.70 ± 0.14) × 10^3^	(3.73 ± 0.96) × 10^3^
	10^2^	(4.31 ± 0.16) × 10^2^	(3.96 ± 0.19) × 10^2^
	10^1^	(5.60 ± 0.65) × 10	(3.80 ± 3.00) × 10
	10^0^	0	8.40 ± 1.08
**COLI**	10^3^	(3.34 ± 0.15) × 10^3^	(4.51 ± 0.42) × 10^3^
	10^2^	(3.67 ± 0.15) × 10^2^	(3.20 ± 0.12) × 10^2^
	10^1^	(4.00 ± 0.50) × 10	(3.80 ± 0.40) × 10
	10^0^	0	3.93 ± 0.75
**TVC**	10^3^	(2.36 ± 0.96) × 10^3^	(3.74 ± 0.67) × 10^3^
	10^2^	(3.19 ± 0.05) × 10^2^	(2.59 ± 0.04) × 10^2^
	10^1^	(4.80 ± 0.66) × 10	(5.70 ± 0.80) × 10^1^
	10^0^	0	7.43 ± 0.18

^a^*E. coli* Escherichia coli*, Sal,* Salmonella*, S. aureus* Staphylococcus aureus*, Listeria* Listeria monocytogenes, *COLI* Coliforms, *TVC* Total viable count

### Comparison of accuracy between the MBS microbial rapid detection method and PCM

The recovery rates of the PCM and MBS rapid microbial detection systems are shown in Table 3. For PMC, the recovery rates of *E. coli, Salmonella, S. aureus,* LM, COLI and TVC were determined: the recovery rates of *E. coli* ranged from 85.58 to 92.60%; the recovery rates of *Salmonella* ranged from 79.77 to 93.48%; the recovery rates of *S. aureus* ranged from 86.49 to 94.01%; the recovery rates of LM ranged from 83.05 to 92.69%; the recovery rates of COLI ranged from 87.44 to 96.58%; and the recovery rates of TVC ranged from 87.09 to 94.98%. For the MBS Microbial Rapid Detection System, the recovery rates of *E. coli, Salmonella, S. aureus,* LM, COLI and TVC were also determined: the recovery rates were 87.92 ~ 98.42%, 87.19 ~ 90.76%, 89.95 ~ 96.32%, 89.15 ~ 92.93%, 90.95 ~ 97.66%, and 90.21 ~ 97.03%, respectively. The recovery rate of the MBS microbial rapid detection system was higher than the recovery rate of the plate-counting method, indicating that the MBS microbial rapid detection system has better accuracy in the detection of the six standard strains in sterilized fish meal. Traversetti et al. [31] reported that the MBS microbial rapid detection system has higher sensitivity, selectivity and precision for the detection of pathogenic microorganisms.

**Table 3:** Comparison of the recovery rates of six standard strains by the two methods

Strains^a^	Bacteria concentration (cfu/mL)	PCM (%)	MBS (%)
***E. coli***	10^8^	85.58 ± 7.22	87.92 ± 8.20
	10^7^	90.07 ± 6.63	95.23 ± 13.24
	10^6^	92.20 ± 8.54	96.61 ± 10.12
	10^5^	92.33 ± 10.99	97.45 ± 13.03
	10^4^	90.85 ± 13.90	88.82 ± 10.21
	10^3^	92.03 ± 15.35	98.42 ± 18.70
	10^2^	92.60 ± 11.81	91.25 ± 17.70
	10^1^	0	0
	10^0^	0	0
***Sal***	10^8^	92.04 ± 6.75	90.76 ± 9.85
	10^7^	90.26 ± 6.12	89.53 ± 5.54
	10^6^	89.16 ± 3.42	87.19 ± 4.71
	10^5^	93.48 ± 8.81	89.98 ± 2.81
	10^4^	91.76 ± 10.18	90.67 ± 3.67
	10^3^	79.77 ± 12.46	87.72 ± 6.24
	10^2^	88.96 ± 6.44	88.60 ± 5.11
	10^1^	0	0
	10^0^	0	0
***S. aureus***	10^8^	86.33 ± 12.63	94.99 ± 4.64
	10^7^	94.01 ± 7.66	91.70 ± 7.40
	10^6^	87.63 ± 8.37	89.95 ± 9.12
	10^5^	95.23 ± 8.86	96.32 ± 6.88
	10^4^	91.26 ± 12.97	95.77 ± 7.49
	10^3^	86.49 ± 16.48	92.82 ± 5.61
	10^2^	90.74 ± 3.42	93.55 ± 5.11
	10^1^	0	0
	10^0^	0	0
***Listeria***	10^8^	92.37 ± 5.20	92.93 ± 5.35
	10^7^	92.69 ± 6.14	91.31 ± 5.61
	10^6^	84.15 ± 6.71	90.46 ± 2.92
	10^5^	90.70 ± 9.70	89.19 ± 5.41
	10^4^	90.86 ± 10.63	92.86 ± 8.93
	10^3^	83.05 ± 10.56	89.15 ± 9.21
	10^2^	83.58 ± 6.79	91.90 ± 9.44
	10^1^	0	0
	10^0^	0	0
**COLI**	10^8^	96.36 ± 6.76	92.59 ± 7.09
	10^7^	96.58 ± 7.41	97.47 ± 7.48
	10^6^	95.06 ± 8.87	97.66 ± 8.11
	10^5^	92.80 ± 7.62	90.95 ± 8.01
	10^4^	87.44 ± 9.79	93.19 ± 3.70
	10^3^	94.08 ± 5.37	96.17 ± 5.83
	10^2^	89.19 ± 6.70	91.68 ± 3.30
	10^1^	0	0
	10^0^	0	0
**TVC**	10^8^	94.17 ± 6.24	95.26 ± 2.61
	10^7^	92.83 ± 7.54	90.21 ± 6.06
	10^6^	94.98 ± 7.15	96.34 ± 6.98
	10^5^	87.09 ± 6.22	94.70 ± 6.69
	10^4^	89.38 ± 5.33	97.03 ± 8.86
	10^3^	90.44 ± 1.42	91.93 ± 2.35
	10^2^	90.17 ± 7.15	91.23 ± 6.34
	10^1^	0	0
	10^0^	0	0

^a^ *E. coli* Escherichia coli*, Sal,* Salmonella*, S. aureus* Staphylococcus aureus*, Listeria* Listeria monocytogenes, *COLI* Coliforms, *TVC* Total viable count

### Comparison of precision between the MBS microbial rapid detection method and PCM

The coefficients of variation of the plate-counting method and MBS rapid microbial detection system are shown in Table 4. The coefficients of variation of *E. coli, Salmonella, S. aureus,* LM, COLI and TVC by the plate-counting method were 14.92, 13.84, 15.63, 12.18, 13.21, and 14.91%, respectively. The coefficients of variation of *E. coli, Salmonella, S. aureus,* LM, COLI and TVC by the MBS Microbial Rapid Detection System were 9.83, 7.18, 10.28, 9.55, 8.03, and 9.21%, respectively. Bottini et al. [23] reported that the CV of *E. coli* and TVC in foodstuffs by the MBS Microbial Rapid Detection System was similar to our results.

**Table 4:** Comparison of the coefficient of variation of six standard strains detected by two methods

Strains^a^	PCM (%)	MBS (%)
***E. coli***	14.92	9.83
***Sal***	13.84	7.18
***S. aureus***	15.63	10.28
***Listeria***	12.18	9.55
**COLI**	13.21	8.03
**TVC**	14.91	9.21

^a^*E. coli* Escherichia coli*, Sal,* Salmonella*, S. aureus* Staphylococcus aureus*, Listeria* Listeria monocytogenes, *COLI* Coliforms, *TVC* Total viable count

### Detection time

The detection times of the two methods are shown in Table 5. From sample processing to data analysis, the detection time for the MBS method for detecting *E. coli, Salmonella, S. aureus,* LM, COLI and TVC with a bacterial concentration of 10^4^ CFU/ml was 11 h, 9 h, 21 h, 17 h, 11 h and 8 h, respectively. The detection times of *E. coli, Salmonella, S. aureus,* LM, COLI and TVC by the PCM method were 25 h, 65 h, 49 h, 49 h, 84 h and 75 h, respectively. As shown in Table 1, the greater the bacterial concentration in the sample, the shorter the detection time required by the MBS method. When the PCM method is used to detect pathogenic bacteria, the isolation and cultivation takes a lot of time, and the steps are complicated, which is not convenient for on-site detection. The MBS method does not need to pretreat the sample; the method is simple and can detect several different pathogens at the same time. Compared with the two methods, the MBS method is easy to use, reliable and simple to interpret, and it is faster and cheaper than traditional detection method [31].

**Table 5:** Comparison of the time required for the two methods to detect six standard strains

**Strains**^a^	**Method**	**Time** **(h)**
***E. coli***	PCM	25
	MBS	11
***Sal***	PCM	65
	MBS	9
***S.aureus***	PCM	49
	MBS	21
***Listeria***	PCM	49
	MBS	17
**COLI**	PCM	84
	MBS	11
**TVC**	PCM	75
	MBS	8

^a^*E. coli* Escherichia coli*, Sal,* Salmonella*, S. aureus* Staphylococcus aureus*, Listeria* Listeria monocytogenes, *COLI* Coliforms, *TVC* Total viable count

### Correlation between the two methods

**Fig. 1:**
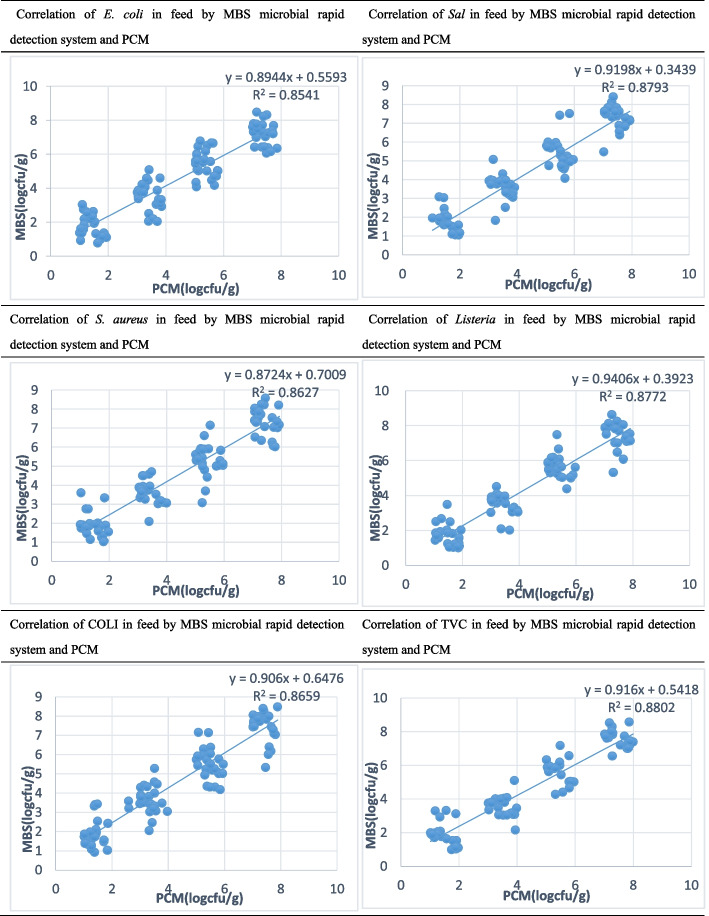
Correlation of pathogenic bacteria in feed by the MBS microbial rapid detection system and PCM

Figure 1 shows that at four concentrations of 10^2^, 10^4^, 10^6^ and 10^8^ cfu/mL, the MBS microbial rapid detection system and the plate-counting method showed a clear linear relationship in the detection of *E. coli, Salmonella, S. aureus,* LM, COLI and TVC in feed, and the R^2^ values were 0.8541, 0.8793, 0.8627, 0.8772, 0.8659 and 0.8802, respectively. Bottini et al. [23] used the MBS rapid microbial detection system and the plate-counting method to detect TVC and *E. coli* in artificially simulated contaminated food samples and found that the detection results of the two methods had a clear linear relationship, and the R^2^ values were 0.94 and 0.99, respectively. Traversetti et al. [31] also used the MBS microbial rapid detection system and plate counting method to detect TVC and *E. coli* in tap water and obtained a linear correlation between the detection results of the two methods, with R^2^ values of 0.74 and 0.72, respectively.

In summary, in this experiment, the MBS microbial rapid detection system and the plate-counting method were used to determine the content of pathogenic bacteria in artificially infected feed, and the analysis of the determination results of the two methods showed that the final determination of the bacteria contents of six different pathogens showed a linear correlation. The microbiological detection results of the MBS microbiological rapid detection system in food and water quality are similar to the detection results of microbes in the feed, indicating that the method is little affected by the matrix in the detection of microorganisms. The MBS microbial rapid detection system can be used as a detection method for pathogenic bacteria in feed.

### Comparison of two methods for the detection of 6 pathogenic bacteria in feed ingredients

As shown in Table 6, *E. coli* was detected only in fish meal and feather meal among the 5 kinds of feed ingredients. The detection rate of *E. coli* in fish meal detected by the two methods was 20%, and there was no significant difference in the mean value of the detection results of the two methods (*P* > 0.05). The detection rate of *E. coli* in feather meal by the two methods was 60%, and there was no significant difference in the mean value of the detection results between the two methods (*P* > 0.05). The detection rate of COLI in blood meal, meat and bone meal, fish meal and feather meal by both methods was 100%, and the detection rate of COLI in whey meal was 20%. There was no significant difference in the mean value of COLI in blood meal, meat and bone meal, fish meal and whey meal detected by the two methods (*P* > 0.05), but the mean value of COLI in feather meal was significantly different (*P* < 0.05). The detection rate of TVC in the feed ingredients detected by the two methods was 100%, and the average value of TVC in the fish meal, feather meal and whey meal detected by the two methods was not significantly different (*P* > 0.05). The mean values of TVC in blood meal and meat and bone meal were significantly different (*P* < 0.05).

**Table 6:** Comparison of the results of detecting pathogenic bacteria in feed ingredients by two methods

Feed ingredient	Strains^a*^	Method	Positive rate (%)	Mean value (μg/kg)	Median value (μg/kg)	Maximum value (μg/kg)
**Blood meal**	*E. coli*	PCM	—	—	—	—
		MBS	—	—	—	—
	COLI	PCM	100	2.82 ± 0.77^a^	2.86	3.90
		MBS	100	2.99 ± 0.60^a^	2.87	3.85
	TVC	PCM	100	3.43 ± 0.83^a^	3.61	4.85
		MBS	100	4.06 ± 0.87^b^	4.44	4.92
**Meat and bone meal**	*E. coli*	PCM	—	—	—	—
		MBS	—	—	—	—
	COLI	PCM	100	2.27 ± 0.70^a^	1.97	3.54
		MBS	100	2.32 ± 0.69^a^	1.94	3.56
	TVC	PCM	100	3.08 ± 0.46^a^	2.96	3.78
		MBS	100	3.03 ± 0.52^b^	2.80	3.87
**Fish meal**	*E. coli*	PCM	20	0.51 ± 0.05^a^	0.48	0.58
		MBS	20	0.46 ± 0.11^a^	0.44	0.59
	COLI	PCM	100	2.35 ± 0.84^a^	1.91	3.79
		MBS	100	2.28 ± 0.76^a^	1.97	3.91
	TVC	PCM	100	3.17 ± 1.07^a^	2.85	5.52
		MBS	100	3.52 ± 0.80^a^	3.50	4.92
**Feather meal**	*E. coli*	PCM	60	2.60 ± 0.26^a^	2.64	2.84
		MBS	60	2.67 ± 0.25^a^	2.76	2.87
	COLI	PCM	100	1.76 ± 0.74^a^	1.93	3.04
		MBS	100	1.92 ± 0.87^b^	2.19	3.16
	TVC	PCM	100	3.37 ± 0.83^a^	3.36	4.91
		MBS	100	3.36 ± 0.79^a^	3.29	4.81
**Whey meal**	*E. coli*	PCM	—	—	—	—
		MBS	—	—	—	—
	COLI	PCM	20	1.87 ± 0.21^a^	1.93	2.12
		MBS	20	1.86 ± 0.20^a^	1.85	2.18
	TVC	PCM	100	2.18 ± 0.57^a^	2.13	2.85
		MBS	100	2.22 ± 0.44^a^	2.19	2.79

^a/b^Means with different superscripts are significantly different within a column (*P* < 0.05). Data are expressed as the mean ± SD (*n* = 5)

^a*^E. coli Escherichia coli*, Sal,* Salmonella*, S. aureus* Staphylococcus aureus*, Listeria* Listeria monocytogenes, *COLI* Coliforms, *TVC* Total viable count

As shown in Table 6, the detection rate of the two methods for detecting COLI in feed mixtures was 100%, and the average value of COLI in broilers complete feed and pig complete feed detected by the two methods was not significantly different (*P* > 0.05). The mean values of COLI in laying hen complete feed and meat duck complete feed were significantly different (*P* < 0.05). The detection rate of the two methods for detecting TVC in feed complete was 100%, and the difference in the mean values was not significant (*P* > 0.05).

When the PCM and the MBS microbial rapid detection systems were used to detect the bacteria in the 9 kinds of feed samples collected, no *Salmonella, S. aureus* or LM were detected. *E. coli* was detected only in fish meal and feather meal. From Tables 6 and 7, the average values of the detection results of *E. coli*, COLI and TVC in individual feed samples by the two methods were found to be significantly different, possibly related to the complex composition of some feeds, and the MBS microbiological rapid detection system has been used mostly in the detection of microorganisms in food before and has not detected microorganisms in the feed, and its internal calibration curve for food may not be completely suitable for the feed. Antonini et al. [32] mentioned in an article on food microbiological safety that when the MBS microbiological rapid detection system is used for microbiological detection in a specific food matrix, due to the interference of the complex composition of the specific food itself and some oxidants, a specific curve calibration must be performed. However, in most feed samples, the average detection values of the two methods are not significantly different, and when the two methods detect the content of the same pathogen, the difference between the median and the highest value of the detection results is not significant, which shows that the overall deviation of the detection results of the two methods is small. This observation is also consistent with the good linear correlation between the two methods in Fig. 1 for the detection of the same pathogen levels. In all feed samples, *Salmonella, Staphylococcus aureus*, and LM were not detected by either method, indicating that the two methods showed good agreement in detecting these pathogens.

**Table 7:** Comparison of the results of detecting pathogenic bacteria in feed mixtures

Feed mixture	Strains^a*^	Method	Positive rate (%)	Mean value (μg/kg)	Median value (μg/kg)	Maximum value (μg/kg)
**Laying hen complete feed**	COLI	PCM	100	2.54 ± 0.74^a^	2.33	4.12
		MBS	100	3.06 ± 0.82^b^	3.01	4.49
	TVC	PCM	100	3.09 ± 0.92^a^	3.09	4.86
		MBS	100	3.08 ± 0.82^a^	3.35	4.36
**broilers complete feed**	COLI	PCM	100	2.43 ± 0.77^a^	2.49	3.72
		MBS	100	2.64 ± 0.78^a^	2.73	3.89
	TVC	PCM	100	3.19 ± 0.59^a^	2.90	3.99
		MBS	100	3.27 ± 0.60^a^	3.60	3.94
**Pig complete feed**	COLI	PCM	100	2.99 ± 0.70^a^	2.89	3.91
		MBS	100	2.98 ± 0.67^a^	2.93	3.86
	TVC	PCM	100	3.54 ± 0.79^a^	3.69	4.88
		MBS	100	3.65 ± 0.87^a^	3.73	4.87
**Meat duck complete feed**	COLI	PCM	100	2.25 ± 0.44^a^	2.19	3.34
		MBS	100	2.63 ± 0.54^b^	2.57	3.68
	TVC	PCM	100	3.47 ± 0.78^a^	3.30	4.85
		MBS	100	3.57 ± 0.66^a^	3.35	4.76

^a/b^Means with different superscripts are significantly different within a column (*P* < 0.05). Data are expressed as the mean ± SD (*n* = 5)

^a*^E. coli Escherichia coli*, Sal,* Salmonella*, S. aureus* Staphylococcus aureus*, Listeria* Listeria monocytogenes, *COLI* Coliforms, *TVC* Total viable count

## Conclusions

When the MBS rapid microbial detection system and the PCM quantitatively detect the content of pathogenic bacteria in artificially contaminated feed, the detection results of the two methods show an obvious correlation. The experiment proves that the MBS microbial rapid detection system can realize the quantitative detection of pathogenic bacteria in feed and shows good accuracy, sensitivity and precision. The MBS microbial rapid detection system does not require pretreatment of feed samples, can directly sample and test on the machine, and has lower requirements for testing personnel and experimental environment, and the detection period is shortened by 2 to 3 times compared with the plate-counting method. The MBS method is convenient, fast and suitable for the detection of pathogenic bacteria on-site in feed.

## Data Availability

The datasets used and/or analyzed during the current study are available from the corresponding author on reasonable request.

## Abbreviations

*S. aureus*: Staphylococcus aureus
LM: Listeria monocytogenes
*E. coli*: Escherichia coli
TVC: Total viable count
COLI: Coliforms
MBS: Micro biology survey
PCM: Plate counting method
ATCC: American type culture collection
CVCC: China veterinary culture collection center
CMCC: Center for medical culture collections
TSB: Trypticase soy broth
LOQ: Limit of Quantitation
